# Impact of abnormal septal geometry on cardiac output in Ebsteins anomaly with tricuspid regurgitation compared to repaired Tetralogy of Fallot

**DOI:** 10.1186/1532-429X-13-S1-P195

**Published:** 2011-02-02

**Authors:** Sylvia SM Chen, Hideki Uemura, Philip J Kilner

**Affiliations:** 1 The Royal Brompton Hospital, London, UK; 2 The Royal Brompton Hospital and Imperial College London, London, UK

## Introduction

We previously found reduction of cardiac index (CI) in patients with tricuspid regurgitation (TR) due to Ebstein anomaly, but not in patients with comparable right ventricular (RV) volume loading due to pulmonary regurgitation (PR) in patients with repaired tetralogy of Fallot (rTOF). We postulated that the difference may be attributable to impaired left ventricular (LV) filling caused by leftward bulging of the atrialized portion of the RV in Ebstein patients.

## Purpose

To measure an index of LV diastolic compression and compare it with CI in different groups.

## Methods

Ebstein and rTOF patients with moderate-severe TR or PR (>20%) measured by cardiovascular magnetic resonance (CMR) without evidence of atrial septal defect or additional haemodynamically significant lesion were included. TR fraction was calculated from RV stroke volume and pulmonary artery forward flow, and PR fraction from pulmonary artery reversed relative to forward flow. The CI was obtained by indexing cardiac output by CMR aortic flow measurement to body surface area. Ventricular volumes were measured from a stack of short-axis cine images. As an index of LV diastolic compression we measured LV width/length at end diastole in 3 chamber long axis cines: the width from septal to lateral wall at basal-mid junction level and the length from mid-mitral valve annulus plane to the LV apex.

## Results

Forty patients, 20 Ebsteins (aged 39±13years, 12 males) and 20 rTOF (aged 27±10years, 11 males) were studied. Sixteen control subjects (32±9years, 9 males) were also studied. Regurgitant fractions in Ebsteins and rTOF were similar, TR 44±18% vs PR 38±12%, p=ns. Compared to controls, CI in Ebsteins was reduced (2.5±0.5L/min/m^2^ vs 3.2±0.5L/min/m^2^, p=0.002) but not in rToF, 3.1±0.6L/min/m^2^, p=ns. LV end-diastolic volume in Ebsteins was less than controls (61±13 vs 75±12ml/m^2^, p<0.001), but comparable in rTOF (81±16ml/m^2^, p=ns). LV ejection fraction was similar between Ebsteins and controls (66±9% vs 69±5%, p=0.4) and lower but within normal range in rTOF (60±9%, p<0.01). RV ejection fraction was decreased in Ebsteins and rToF (48±12% and 49±7% vs controls 56±7%, both p<0.001). LV width/length index was smaller in Ebsteins than controls (0.4±0.1 vs 0.5±0.1, p=0.004) but not in rTOF (0.5±0.1, p=ns). LV end-diastolic volume correlated with LV width/length index (r^2^=0.4) and CI (r^2^=0.6) in Ebstein**.**

## Conclusions

The findings support the hypothesis that impairment of LV filling by leftward displacement of the septum contributes to the reduction of CI found in Ebstein patients.

